# Genetic polymorphism of *Plasmodium falciparum* circumsporozoite protein on Bioko Island, Equatorial Guinea and global comparative analysis

**DOI:** 10.1186/s12936-020-03315-4

**Published:** 2020-07-13

**Authors:** Hui-Ying Huang, Xue-Yan Liang, Li-Yun Lin, Jiang-Tao Chen, Carlos Salas Ehapo, Urbano Monsuy Eyi, Jian Li, Ting-Ting Jiang, Yu-Zhong Zheng, Guang-Cai Zha, Dong-De Xie, Jin-Quan He, Wei-Zhong Chen, Xiang-Zhi Liu, Huan-Tong Mo, Xin-Yao Chen, Min Lin

**Affiliations:** 1 School of Food Engineering and Biotechnology, Hanshan Normal University, Chaozhou, People’s Republic of China; 2 Department of Medical Genetics, Shantou University Medical College, Shantou, Guangdong Province People’s Republic of China; 3 Department of Medical Laboratory, Huizhou Central Hospital, Huizhou, Guangdong Province People’s Republic of China; 4 The Chinese Medical Aid Team to the Republic of Equatorial Guinea, Guangzhou, Guangdong Province People’s Republic of China; 5 Department of Medical Laboratory, Malabo Regional Hospital, Malabo, Equatorial Guinea; 6 Department of Human Parasitology, School of Basic Medical Sciences, Department of Infectious Diseases, Renmin Hospital, Hubei University of Medicine, Shiyan, Hubei Province People’s Republic of China; 7 Department of Medical Laboratory, Chaozhou People’s Hospital Affiliated to Shantou University Medical College, Chaozhou, Guangdong Province People’s Republic of China

**Keywords:** Malaria, *Plasmodium falciparum*, Circumsporozoite protein, Genetic polymorphism, Bioko Island

## Abstract

**Background:**

*Plasmodium falciparum* circumsporozoite protein (PfCSP) is a potential malaria vaccine candidate, but various polymorphisms of the *pfcsp* gene among global *P. falciparum* population become the major barrier to the effectiveness of vaccines. This study aimed to investigate the genetic polymorphisms and natural selection of *pfcsp* in Bioko and the comparison among global *P. falciparum* population.

**Methods:**

From January 2011 to December 2018, 148 blood samples were collected from *P. falciparum* infected Bioko patients and 96 monoclonal sequences of them were successfully acquired and analysed with 2200 global *pfcsp* sequences mined from MalariaGEN Pf3k Database and NCBI.

**Results:**

In Bioko, the N-terminus of *pfcsp* showed limited genetic variations and the numbers of repetitive sequences (NANP/NVDP) were mainly found as 40 (35%) and 41 (34%) in central region. Most polymorphic characters were found in Th2R/Th3R region, where natural selection (*p *> 0.05) and recombination occurred. The overall pattern of Bioko *pfcsp* gene had no obvious deviation from African mainland *pfcsp* (Fst = 0.00878, *p *< 0.05). The comparative analysis of Bioko and global *pfcsp* displayed the various mutation patterns and obvious geographic differentiation among populations from four continents (p < 0.05). The global *pfcsp* C-terminal sequences were clustered into 138 different haplotypes (H_1 to H_138). Only 3.35% of sequences matched 3D7 strain haplotype (H_1).

**Conclusions:**

The genetic polymorphism phenomena of *pfcsp* were found universal in Bioko and global isolates and the majority mutations located at T cell epitopes. Global genetic polymorphism and geographical characteristics were recommended to be considered for future improvement of malaria vaccine design.

## Background

Malaria, caused by *Plasmodium* spp. infections, is one of the most significant life-threatening infectious diseases to humans worldwide. According to the World Malaria Report 2019 [1], an estimated 228 million (95% confidence interval [CI] 206–258 million) persons suffered from malaria infections worldwide, with 405,000 malaria deaths in 2018. Twenty countries accounted for 85% of global malaria cases in 2018; all these countries are in sub-Saharan Africa, except for India. Resistance to anti-malarial drugs and insecticides, coupled with the lack of availability of an effective vaccine, is the leading factors behind the parasite’s continuing burden. Apart from its complex life cycle, which alternates between the human and the mosquito host, the malaria parasite also exhibits stages characterized by extensive genetic and antigenic diversity which may present adverse obstacles to anti-malarial control measures.

Currently, there are many efforts and studies have been performed in order to develop effective vaccines, several potential vaccine candidates targeted against pre-erythrocytic, erythrocytic and sexual stages of *Plasmodium falciparum* are under various stages of clinical development [2, 3]. RTS, S/AS01 vaccine is a pre-erythrocytic stage vaccine based on the *P. falciparum* circumsporozoite protein (PfCSP) [4, 5]. In 2015, the European Medicines Agency for the immunization of children against malaria approved the RTS, S/AS01 vaccine [6] and the phase 3 clinical trials conducted in various sites in Africa showed that the RTS, S/AS01 vaccine has a protective efficacy of 45% in children in the first twenty months after vaccination [7, 8]. In 2018, the World Health Organization through a large-scale pilot malaria vaccine implementation program (MVIP) aimed to introduce this vaccine in three sub-Saharan countries (Ghana, Kenya, Malawi) [6]. Besides of RTS, S/AS01, a live attenuated *Plasmodium falciparum* whole sporozoite (SPZ) vaccine is also regarded as a great potential malarial vaccine. Sanaria^®^ PfSPZ Vaccine had conducted a clinical trial on Bioko Island where 70% vaccinees developed antibodies to *P. falciparum* circumsporozoite protein, which was the first clinical trial conducted in Equatorial Guinea [9]. It is not hard to see that *pfcsp* is a very important gene for the host immune response to the *P. falciparum* invasion.

PfCSP is predominantly distributed on the surface of the sporozoites with a molecular mass of about 58 kDa. PfCSP is GPI-anchored on the sporozoite surface and plays a critical role in sporozoite development, motility and hepatocyte invasion [10, 11]. The structure of PfCSP can be divided into three distinct regions: a highly variable central repeat region flanked by a conserved N-terminal region and a C-terminal non-repeat region [12]. The central repeat region, which has been recognized as a major target for antibody-mediated neutralization, is rich in Asn-Ala-Asn-Pro (NANP) tandem repeats and contains a small number of Asn-Val-Asp-Pro (NVDP) motifs [12], constitutes immunodominant B cell epitopes. The C-terminal non-repeat region includes two polymorphic sub-regions, Th2R and Th3R, where T cell epitopes were identified.

The previous studies revealed higher single nucleotide polymorphisms (SNPs) of *pfcsp* within the *P. falciparum* population from different geographic regions [13]. Indeed, most *P. falciparum* vaccine candidate gene including *pfcsp* have been found to show various genetic and antigenic polymorphisms in global parasites, which might obstruct or reduce the efficacy of vaccines [14, 15].

Understanding the genetic nature of vaccine candidate antigens is critical for designing an effective vaccine. The aims of the present study are to investigate the polymorphism pattern of *pfcsp* gene and its diversifying selection of *P. falciparum* on Bioko Island, and to elucidate how *pfcsp* gene is differentiated among global *P. falciparum* populations. This study will fill in the blank of Bioko Island *pfcsp* data, as well as be helpful not only for understanding the molecular evolution of the *pfcsp* gene in *P. falciparum,* but also for designing peptide-based vaccines for the PfCSP antigen.

## Methods

### Study area

The study was carried out in Malabo Regional Hospital and the clinic of the Chinese medical aid team to the Republic of Equatorial Guinea. Bioko is an island 32 km off the west coast of Africa and located in the northernmost part of Equatorial Guinea. The island has a population of 334,463 (2015 census), of which approximately 90% live in Malabo (the capital city of Equatorial Guinea) in a humid tropical environment. Malaria due to *P. falciparum* is a major public health problem on the island [16]. Since the Bioko Island Malaria Control Project (BIMCP) has launched at 2004, the parasite prevalence on Bioko decreased from over 45% prevalence in 2004 to 8.5% in 2016, and the reduction of entomological inoculation rate from more than 1000 before 2004 to 14 in 2015 (www.mcdinternational.org).

### Ethical approval

Verbal informed consent was obtained from all participating subjects or their parents, and this study, as well as the consent process, was approved by the Ethics Committee of Malabo Regional Hospital. The Ethical approval letter had been shown as Additional files 1 and 2.

### Samples collection

A total of 148 blood spot samples were collected from the patients with uncomplicated malaria during January 2011–December 2018 in Bioko Island. Included patients were residents on Bioko Island aged between 4 months and 80 years. Malaria patients were classified into uncomplicated malaria states according to the WHO criteria, which were defined as positive smear for *P. falciparum* and presence of fever (≥ 37.5 °C). Dried blood spots were collected on day zero of enrollment through finger prick bleeding spotted onto Whatman 903^®^ filter paper (GE Healthcare, Pittsburgh, USA) for future use. Laboratory screening for malaria was done using rapid diagnostic tests (RDT) and confirmed using microscopic examination of blood smears. For quality control, archived malaria-positive microslides were re-examined and parasite density was recorded. The *Plasmodium* species was identified by a real-time PCR followed by high-resolution melting (HRM) [17]. The pGEM-T standard plasmids of four human *Plasmodium* species including *P. falciparum*, *Plasmodium ovale*, *Plasmodium malariae* and *Plasmodium vivax*, which were kindly provided by Dr. Cao (Jiangsu Institute of Parasitic Diseases, Wuxi, Jiangsu Province, China), were used as control.

### Genomic DNA extraction

Parasite genomic DNA was extracted from dried filter blood spots by Chelex-100 extraction method described in previous article [18]. The DNA products were collected in sterile tubes and stored at − 80 °C in reserve.

### Amplification of the entire *pfcsp* gene

The entire *pfcsp* gene (NCBI Gene ID: 814364) was amplified by nested PCR. For the first round PCR, 2μl of genomic DNA was amplified with 0.25μl 2× HotStart DNA Polymerase, 2μl dNTP Mixture, 5μl 5× PCR buffer, 1μ1 10 mol/L forward primer (5′-CCGGTCATAAATTCTGAATTATCAA-3′), 1μl 10 mol/L reverse primer (5′-CTACAATTAATCGCAAACGTA-3′), and sterile ultra-pure water to a final volume of 25μl. Thermal cycling parameters for PCR were as follows: initial denaturation at 95 °C for 3 min; 30 cycles of 98 °C for 10 s and 68 °C for 90 s. For the second round PCR, 3μl of the primary PCR product was amplified in a 50μl reaction volume comprised of 0.4μl HotStart DNA Polymerase, 3.2μl dNTP Mixture, 8μl 5 × PCR buffer), 1.6 μl 10 mol/L forward primer (5′-CGTGTAAAAATAAGTAGAAA CCACG-3′), 1.6 μl 10 mol/L reverse primer (5′-GTACAACTCAAACTAAG ATGTGTTC-3′), and sterile ultra-pure water to a final volume of 50μl. PCR procedure was as follows: initial denaturation at 95 °C for 3 min; 30 cycles of 98 °C for 10 s and 68 °C for 90 s. All PCR products were analysed using 1.2% agarose gel electrophoresis, and then, they were purified and sequenced by using ABI 3730×L automated sequencer (Shanghai Yingjun Biotechnology Co., LTD, Guangzhou branch). To ensure the accuracy of the sequencing, at least two clones for each isolate were sequenced. Sequencing primers were the reverse primers of the second round PCR; all the sequences were analysed and integrated by MEGA 6.0 software [19].

### Sequences analysis

The *pfcsp* sequence of the laboratory-adapted *P. falciparum* strain 3D7 (NCBI Gene ID: 814364) was included in the alignment for comparison as a reference sequence. The values of segregating sites (S), number of Haplotypes (H), haplotype diversity (Hd), and observed average pairwise nucleotide diversity (π) were calculated using DnaSP version 6.12.01 [20]. The π was also calculated on a sliding window plot of 10 bases with a step size of 5 bp in order to estimate the stepwise diversity across the sequences. In order to test the null hypothesis of neutrality of *pfcsp*, the rates of synonymous (dS) and nonsynonymous (dN) substitutions were estimated and were compared by MEGA 6.0 program using Nei and Gojobori’s method [21] with the Jukes and Cantor (JC) correction of 1000 bootstrap replications. Tajima’s D test [22], Fu and Li’s D and F statistics analysis [23] were performed using DnaSP 6.12.01 in order to evaluate the neutral theory of natural selection (Table 1). The recombination parameter (R), which included the effective population size and probability of recombination between adjacent nucleotides per generation, and the minimum number of recombination events (Rm) were analysed using DnaSP 6.12.01 (Table 1).

**Table 1:** Genetic diversity and natural selection test and recombination analysis of global *PfCSP* C-terminus

	S	H	Hd ± SD	dN-dS	Tajima’D	Fu and Li’s D	Fu and Li’s F	Ra	Rb	Rm
Bioko island	33	34	0.962 ± 0.008	0.0166	− 0.68556	− 1.23926	− 1.22255	0.0888	32.1	8
Cameroon	12	7	0.944 ± 0.70	0.037	− 0.84239	− 0.68964	− 0.81319	0.0919	15.9	2
Gambia	23	21	0.921 ± 0.022	0.0182	0.13689	0.31057	0.29579	0.057	21.5	6
Ghana	37	89	0.960 ± 0.004	0.0194	− 0.48482	0.03757	− 0.23777	0.0897	33.8	7
Senegal	31	42	0.954 ± 0.008	0.0193	− 0.15853	0.44100	0.23957	0.0992	37.4	6
Tanzania	21	29	0.966 ± 0.009	0.059	0.60634	0.80724	0.875	0.1838	31.8	6
Congo	22	25	0.947 ± 0.011	0.0175	0.18825	0.17445	0.21407	0.0666	25.1	6
Nigeria	7	4	0.900 ± 0.161	− 0.194	0.13160	0.78960	0.69173	0.1954	33.8	0
Mali	23	26	0.947 ± 0.014	0.0192	0.16275	0.90715	0.76259	0.1069	40.3	5
Malawi	28	51	0.965 ± 0.004	0.0189	0.26223	0.56701	0.52963	0.0883	33.3	9
Guinea	23	26	0.955 ± 0.011	0.0185	0.08885	1.17263	0.93102	0.1271	47.9	5
India	2	2	0.389 ± 0.164	0.0057	0.19590	1.063	0.94854	0.0006	0.1	0
Iran	2	2	0.467 ± 0.075	0.0068	1.56386	0.85807	1.20551	0.0127	2.2	0
Myanmar	7	7	0.342 ± 0.080	0.0021	− 1.06145	0.43133	− 0.06134	0	0.001	1
Philippines	18	9	0.821 ± 0.041	0.073	1.01981	1.64995*	1.70088*	0.0977	16.9	1
Thailand	13	9	0.535 ± 0.048	0.0066	− 0.16926	0.24690	0.11276	0	0.001	4
Vietnam	18	11	0.757 ± 0.034	0.0122	− 0.03642	0.81913	0.60286	0.009	3.4	3
Bangladesh	20	14	0.890 ± 0.036	0.0134	− 0.53740	− 0.45410	− 0.56526	0.0549	20.7	5
Cambodia	22	22	0.812 ± 0.009	0.0115	0.10655	1.81010*	1.33256	0.0231	8.7	6
Laos	18	10	0.813 ± 0.030	0.0108	− 0.13493	1.27356	0.91834	0.0154	5.8	3
Brazil	8	3	0.459 ± 0.080	0.032	0.72190	1.30884	1.31767	0	0.001	0
Venezuela	14	7	0.911 ± 0.077	0.023	0.64552	0.79924	0.85743	0.167	28.9	2
PNG	5	4	0.532 ± 0.034	0.02	0.41918	− 0.01266	0.15105	0.0283	4.9	1
Solomon	4	3	0.405 ± 0.069	0.031	− 0.00704	1.00249	0.80842	0	0.004	0
Vanuatu	2	2	0.276 ± 0.046	0.016	0.70558	0.67407	0.80111	0	0.001	0

n: number of samples; S: segregating sites; H: number of haplotypes; Hd: haplotype diversity; dN: the number of synonymous substitutions per site; dS: the number of nun-synonymous substitutions per site; Ra: estimate of recombination between adjacent sites; Rb: estimate of recombination per gene; Rm: minimum number of recombination events; *p < 0.05

### Sequence acquisition and global analysis

The genetic diversities of *pfcsp* among global *P. falciparum* isolates were analysed. A total of the 2200 *pfcsp* sequences from 24 countries or areas were acquired as follows: (i) 1747 monoclonal sequences of Bangladesh, Cambodia, Congo, Gambia, Ghana, Guinea, Laos, Malawi, Mali, Myanmar, Nigeria, Senegal, Thailand and Vietnam were extracted successfully by mining the MalariaGEN Pf3k Project (release 5) [13] using samtools [24] and vcftools [25]; (ii) 453 sequences of Philippines, Iran, India, Papua New Guinea (PNG), Vanuatu, Solomon Islands, Cameroon, Tanzania, Venezuela and Brazil were obtained from NCBI database (Additional file 3). Genetic polymorphism and tests of neutrality were calculated for each population using DnaSP 6.12.01 and MEGA 6.0 as described above. A logo plot was constructed for each *pfcsp* population using the WebLogo program (https://weblogo.berkeley.edu/logo.cgi). In order to investigate the genetic relationships among global *pfcsp* haplotypes, the haplotype network for C-terminal of *pfcsp* from Bioko and other 24 countries and areas listed above was constructed by Popart program (http://popart.otago.ac.nz) using Median-Joining method [26].

### Prediction of impact of amino acid change upon protein structure

The crystallized structure of PfCSP C-terminus, PDBID 3VDK [27] was applied in analysis. PolyPhen-2 [28] and SIFT [29] online serve was used to predict potential impact of amino acid substitutions on the structure or function. Using FOLDX plugin [30] in YASARA [31] to predict the changes in free energy before and after the mutations: ΔΔG(change) = ΔG(mutation) − ΔG(wild-type). As a ‘rule of thumb’: ΔΔG (change) > 0: the mutation is destabilizing; ΔΔG (change) < 0: the mutation is stabilizing.

## Results

### Amplification of Bioko *pfcsp*

Of the 148 blood samples extracted from the collections in Bioko Island, 118 yielded suitable *pfcsp* amplicons for sequencing. Finally, 96 full-length monoclonal *pfcsp* were analysed in this study and 22 polyclonal *pfcsp* were excluded. As expected, size variations were observed in the amplified *pfcsp* sequences. The approximate sizes of amplified products varied from 1.1 to 1.2 kb, which was mainly caused by differences in the number of tandem repeats in the central repeat region. These nucleotide sequences have been deposited at GenBank under Accession Numbers (MN623126–MN623221).

### Genetic polymorphisms of N-terminal region of Bioko and global *pfcsp*

**Fig. 1:**
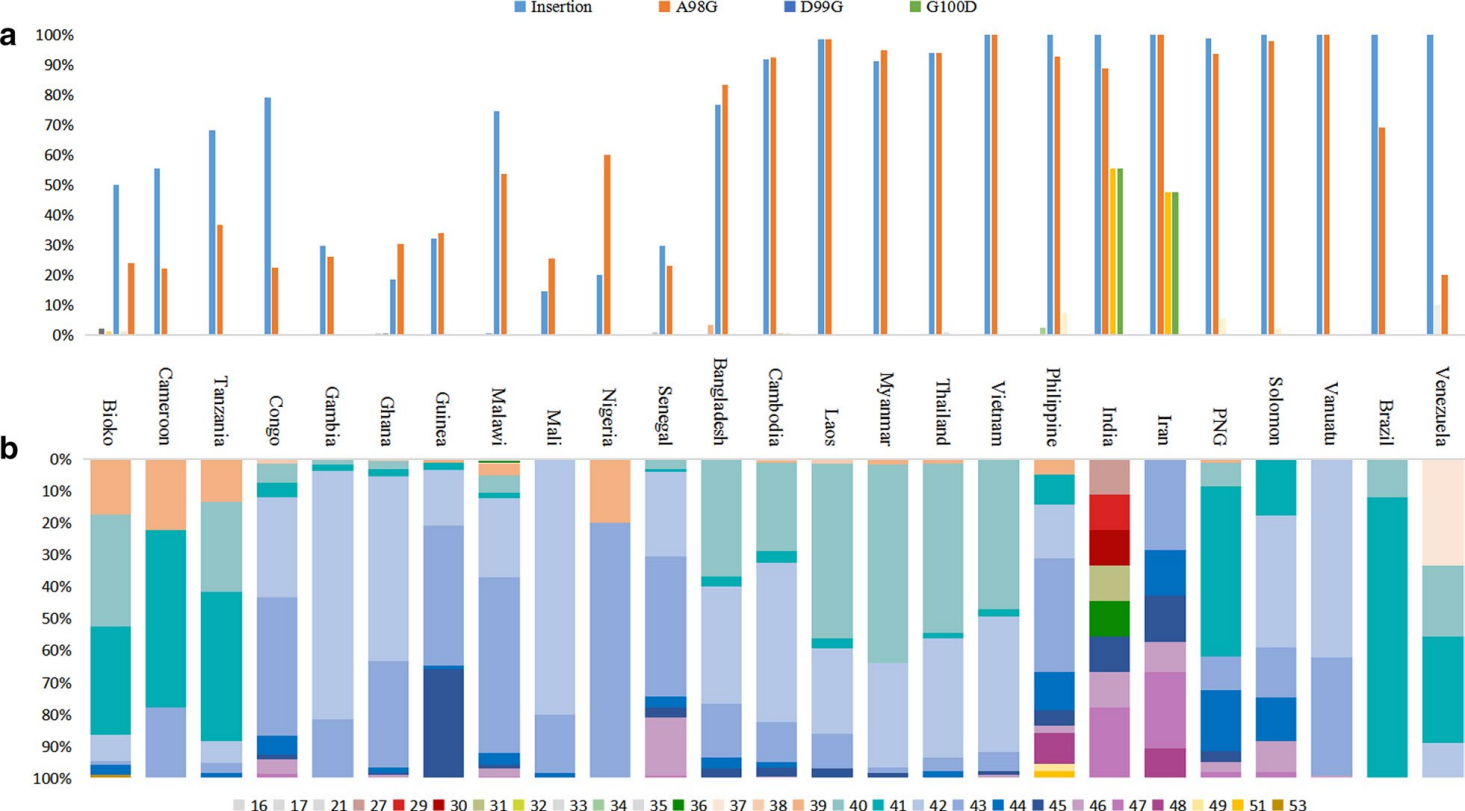
Global sequence polymorphism of *pfcsp* N-terminus and central repeat region. **a** Mutation type and frequency of N-terminal region of global *pfcsp*. Mutations with frequency < 10% were not marked. **b** Frequencies composition of NANP/NVDP repeat numbers in central repeat region among global *pfcsp*

The N-terminal non-repeat region was relatively conserved in Bioko *pfcsp*. Compared with the 3D7 reference sequence (XM_001351086), five variations were found in *pfcsp* N-terminal region of Bioko parasites including L5F (2.08%, 2/96), R70K (1.04%, 1/96), D82N (1.04%, 1/96), A98G (24%, 23/96) and a 57 bp (encoding 19 amino acids of ^80^NNGDNGREGKDEDKRDGNN^81^) insertion (50%, 48/96). A comparative analysis of the N-terminal non-repeat region in global *pfcsp* also showed that the region is relatively well-conserved in global parasites. As shown in Fig. 1a, the 19 amino acids length insertion and A98G were two major variations observed in global *pfcsp*. Almost all Asian and Oceanian countries showed a high frequency of insertion and A98G (ranging from 80 to 100%), but lower in African and American isolates (ranging from 15 to 79%). Meanwhile, some variations showed uneven geographic distributions and in relatively low frequencies. As shown in Fig. 1a, D99G and G100D were only detected from about 50% of Indian and Iranian parasites.

### Genetic polymorphisms of central repeat region of Bioko and global *pfcsp*

A total of 7 haplotypes of Bioko *pfcsp* central region was found at amino acid levels (Fig. 1b). The number of NANP/NVDP repeats were analysed and compared among Bioko and global isolates. In Bioko *pfcsp*, the number of repetitive sequences (NANP/NVDP) were mainly found as 40 (35%, 34/96) and 41 (34%, 33/96). Globally, the number of NANP/NVDP repeat were differed by geographic location. As shown in Fig. 1b, repeat number of majority global isolates in this study were ranging from 40 to 43, while the patterns of Philippines, India and Iran were more polymorphic than others.

### Genetic polymorphisms and natural selection of the C-terminal non-repeat region in Bioko and global *pfcsp*

**Fig. 2:**
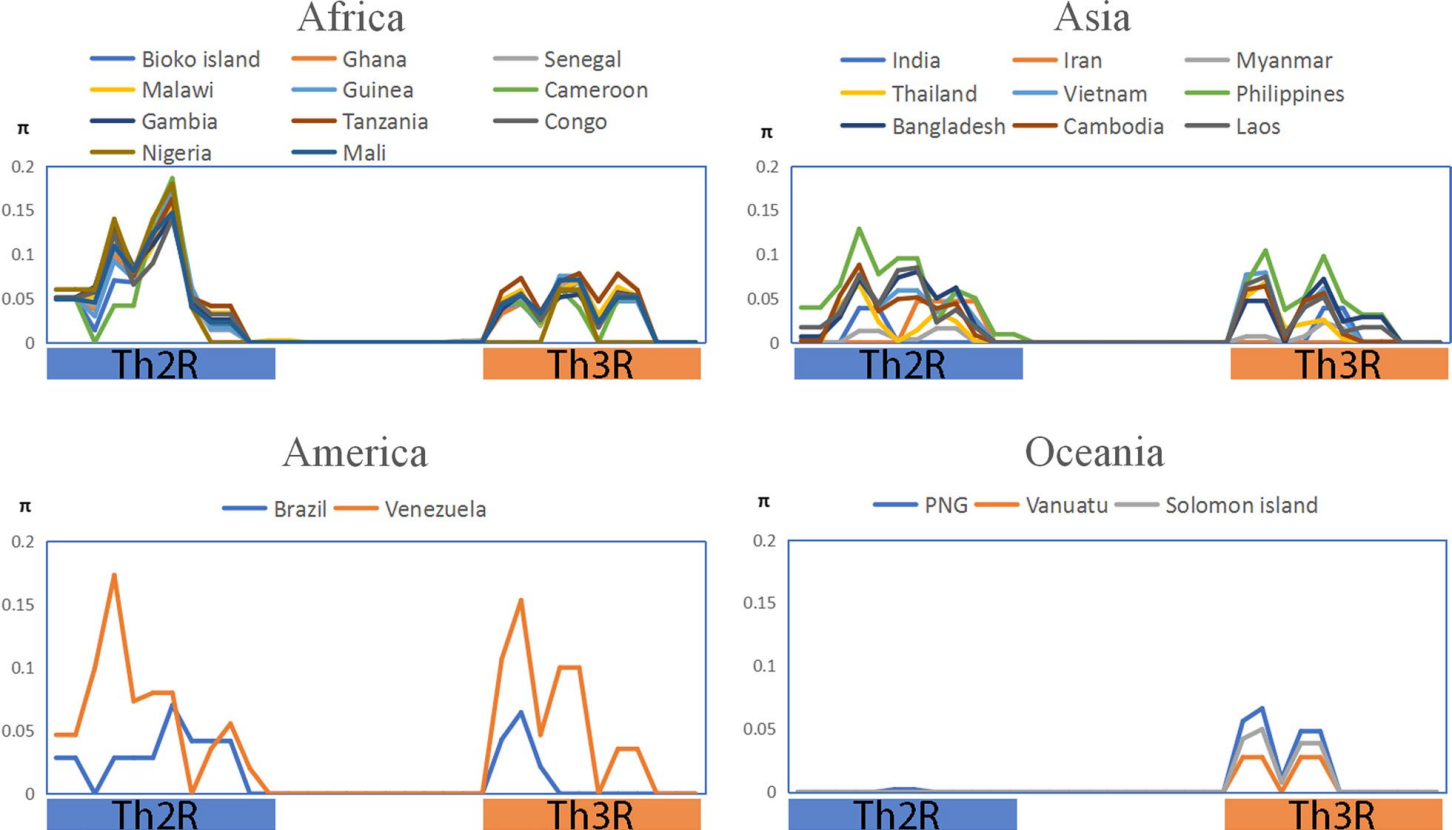
Global nucleotide diversity (π) of C-terminal (311–363) region. The values of nucleotide diversity (π) were calculated using DNASP version 6.12.01 with the sliding window length of 10 bp and step size of 5 bp

Nucleotide diversity (π) of the C-terminal non-repeat region was analysed in Bioko and global *pfcsp* (Fig. 2). Both Th2R (^314^KHIKEYLNKIQNSL^327^) and Th3R (^352^NKPKDELDYAND^363^) region, the proven T cell epitopes, are in high nucleotide diversity, while the connecting region between Th2R and Th3R was conserved. The pattern of nucleotide diversity in Bioko *pfcsp* was perfectly matched with other African countries ones. Compared to patterns of Asia, Africa and America, the one of Oceania was in relatively low diversity, especially in Th2R region, which nearly shows no nucleotide diversity (Fig. 2).

The parameters associated with nucleotide diversity and natural selection were also evaluated on C-terminus non-repeat region (311–363) of Bioko and global *pfcsp* (Table 1). The average number of nucleotide diversity (K) of Bioko *pfcsp* was 5.775 and the overall haplotype diversity (Hd) was 0.962 ± 0.008. The estimated value of dN-dS in Bioko *pfcsp* was found to be 0.0166 (Table 1). For further analysis of natural selection in the C-terminus of Bioko *pfcsp*, Tajima’s test and Fu and Li’s test were performed and the result was shown in Table 1. Both Tajima’s D (− 0.68556, *p *> 0.1) and Fu and Li’s F and D (− 1.23926, *p *> 0.1 and − 1.22255, *p *> 0.1, respectively) values were found to be negative.

As for globally situation, Hd of African countries were generally higher than others (Hd > 0.9), which verified the higher level of genetic diversity on African *pfcsp*. The global dN-dS were shown as positive except Nigeria, and global Tajima’s D values were deviation from 0 in different extents. Recombination events were also evaluated among both Bioko and global *pfcsp*. As shown in Table 1, relative high recombination parameters were shown in all African countries and Philippines, Bangladesh and Venezuela, while lower recombination parameters in other countries.

**Fig. 3:**
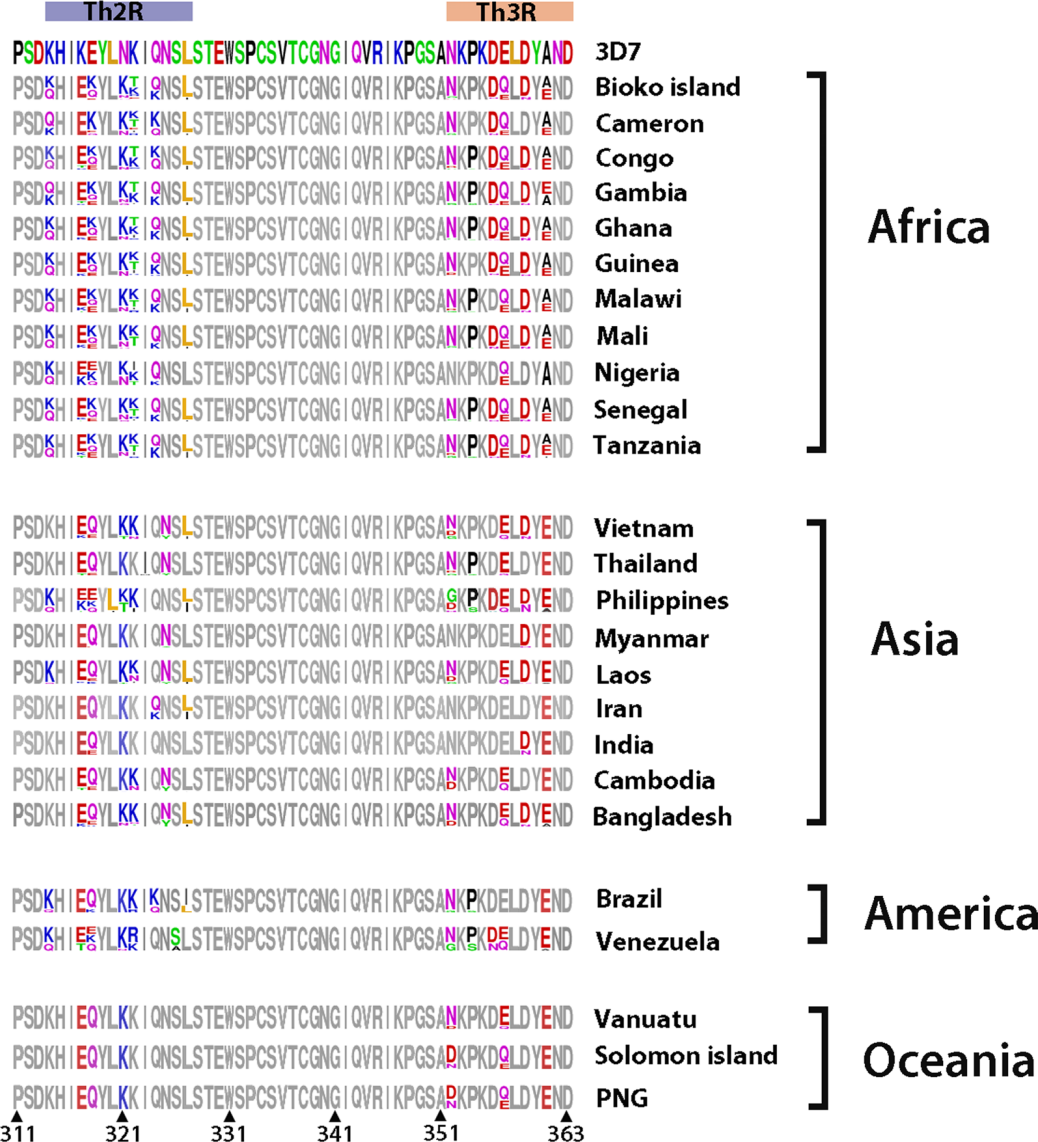
Non-synonymous mutation in C-terminus (311–363) of global *pfcsp*. Each logo consists of stacks of symbols, one stack for each position in the sequence. The height of the amino acid abbreviation indicates its relative frequency at that position. As for the pattern of 3D7 isolate, all positions were marked in coloured; as for global *pfcsp* patterns, mutation sites are marked in colour, while the conserved sites are in gray

In terms of amino acid, the mutation types and its frequencies in C-terminus (311–363) were briefly presented in Fig. 3. There were totally 26 logos generated, one for 3D7 reference isolate and 25 for isolates from different countries and areas. As for Bioko *pfcsp*, mutations were detected at twelve positions (314, 317, 318, 321, 322, 324, 327, 352, 356, 357, 359, 361). All these positions were situated at two T-cell epitopes (Th2R and Th3R). The overall pattern of Bioko is similar to those of African countries. Relatively, more kinds of mutations existed in African isolates, as well as in Philippine and Venezuelan isolates. In contrast, the Oceanian mutation patterns were tended to more uncomplicated. Rare mutation L320I was only found in Philippines while S326A was only found in Venezuela. The high frequency mutation, A361E, existed in all 25 countries, while its wild type (A361) was mainly found in Africa. Notably, the wild type residues of 317, 318, and 321 positions were rarely seen in global isolates, instead, K317E, E318K, E318Q, N321K were mainly found in these positions (Fig. 3).

### Mutation distribution and C-terminus point mutation effect prediction

By analysing with global data, a total of 66 amino acid substitutions were found in the full-length *pfcsp* sequences. In order to know about the distribution of T cell epitopes of *pfcsp*, the proven epitopes (CD8+ and CD4+) were searched from IEDB database [32–39].

**Fig. 4:**
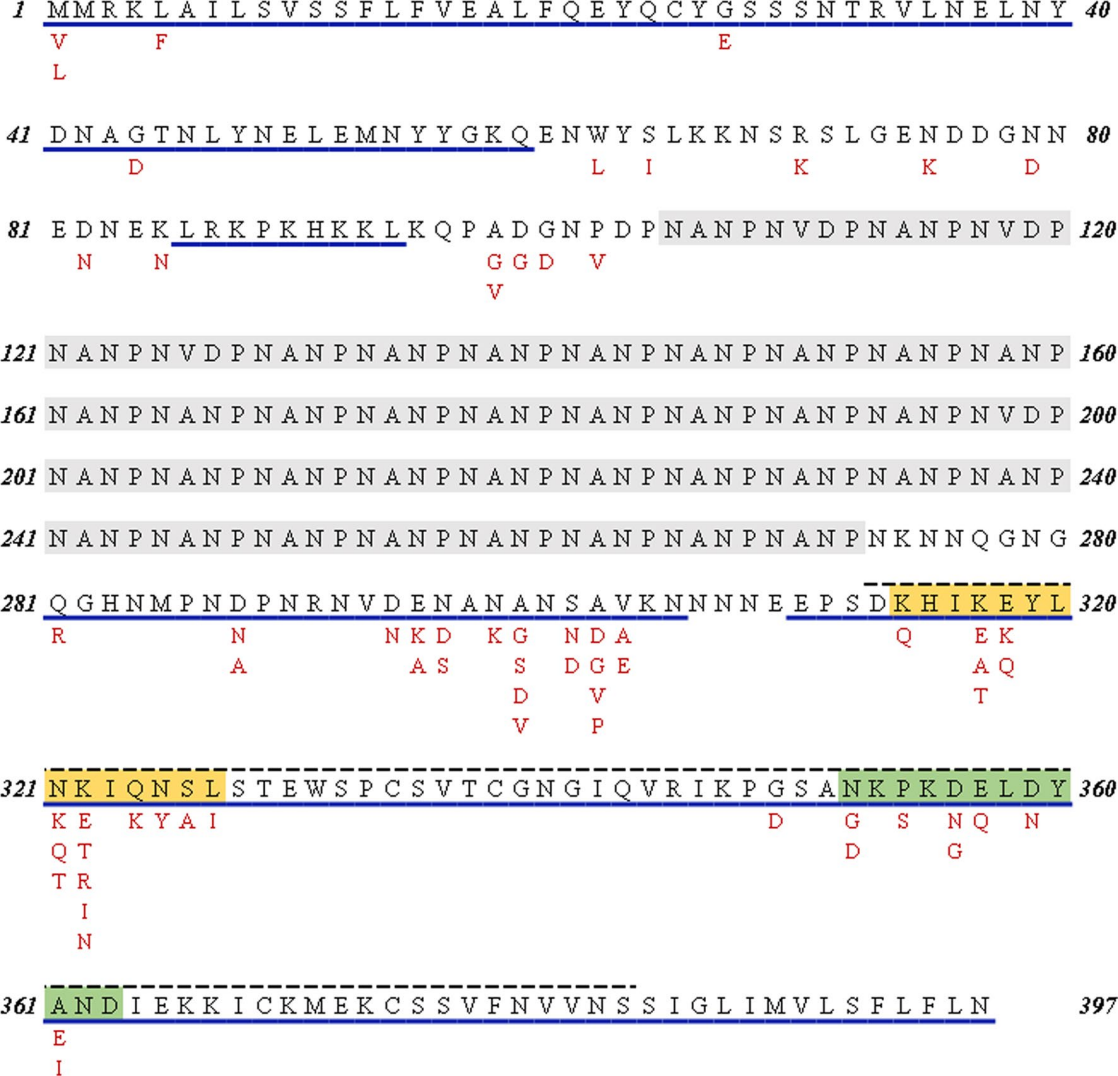
Mutations distribution and T cell epitopes map of *pfcsp* (3D7 isolate). Capital letters in black are amino acid sequences of 3D7 isolate; The red capital letters under the black ones are for mutants. Sequences with black solid line below indicated CD8+ T cell epitopes, sequences with blue dotted line above indicated CD4+ T cell epitopes. Repeat region is in gray shadow; Th2R region is in orange shadow; Th3R region is in green shadow

As shown in Fig. 4, 54 mutations were distributed in T cell epitopes. Majority mutations (74%) were located at the C-terminus of *pfcsp*, as well as the CD8+ T cell epitopes. Notably, there were 28 variances found in the TSR region (including Th2R and Th3R), which also is the overlap of CD4+ and CD8+ T cell epitopes. Furthermore, mutation effect prediction was conducted among these 28 variances. As shown in Table 2, the mutations K322I, N325Y and S326A were predicted to be deleterious using SIFT program (SIFT < 0.05). According to Humdiv score predicted by PolyPhen 2.0 program, 13 mutants were predicted as benign, 4 mutants were possibly damaging and 11 for probably damaging. Among these probably damaging mutants, the protein structures of K317T, K317A, L327I, N352G, P354S and A361I were tending to destabilize (ΔΔG > 0). Some high frequency mutations such as K317E (84.32%), N321K (84.76%) and A361E (72.43%), were predicted as benign. Some extremely low frequency but predicted damaging mutations like K317A (0.17%), S326A (0.09%), G349D (0.13%) and D356G (0.09%), were lack of persuasion (Table 2).

**Table 2:** Global PfCSP C-terminal Mutant types and effect prediction

Mutant	Frequency in global (X/2296)	Frequency in Bioko (X/96)	SIFT	Humdiv	ΔΔG
K314Q	21.78%	47.92%	0.13	0.997A	− 0.373664
K317E	84.32%	93.75%	0.43	0.146C	1.07621
K317T	6.97%	1.04%	0.2	0.995A	1.20284
K317A	0.17%	0	0.08	0.988A	1.31391
E318K	23.52%	56.25%	0.08	0.04C	1.29697
E318Q	59.67%	34.38%	0.06	0.02C	0.981937
N321K	84.76%	79.17%	0.84	0.043C	− 1.74619
N321Q	1.83%	6.25%	0.54	0.954A	− 0.111155
N321T	2.92%	0	0.51	0.082C	− 1.89854
K322E	1.61%	8.33%	0.09	0.186C	0.882041
K322I	5.97%	5.21%	0.02*	0.353C	− 0.667251
K322R	4.44%	10.42%	0.16	0.521B	0.504493
K322T	16.9%	43.75%	0.06	0.186C	1.11661
K322N	6.45%	0	0.06	0.186C	1.04753
Q324K	19.29%	39.58%	0.29	0.043C	− 0.294208
N325Y	7.58%	0	0.02*	0.186C	0.241653
S326A	0.09%	0	0.04*	0.94B	− 0.00308024
L327I	11.06%	10.42%	0.82	0.994A	1.56456
G349D	0.13%	0	0.83	0.999A	− 0.608967
N352G	8.36%	9.38%	0.14	0.994A	0.117207
N352D	18.73%	6.25%	0.08	0.523B	0.0203338
P354S	4.66%	1.04%	0.18	0.988A	1.85106
D356N	3.79%	9.38%	0.1	0.995A	− 0.222874
D356G	0.09%	2.08%	0.09	0.988A	0.0325801
E357Q	45.78%	76.04%	0.13	0.723B	− 0.334979
D359N	8.84%	14.58%	0.2	0.288C	0.433524
A361E	72.43%	46.88%	0.79	0.186C	0.273329
A361I	0.96%	1.04%	0.46	0.973A	0.206936

* SIFT < 0.05, predicted to be deleterious; HumdivA > = 0.953, probably damaging; 0.953 > HumdivB > = 0.432, possibly damaging; 0.432 > HumdivC > = 0.0024, benign

### Population differentiation analysis of *pfcsp* C-terminus among global *P. falciparum* isolates

**Fig. 5:**
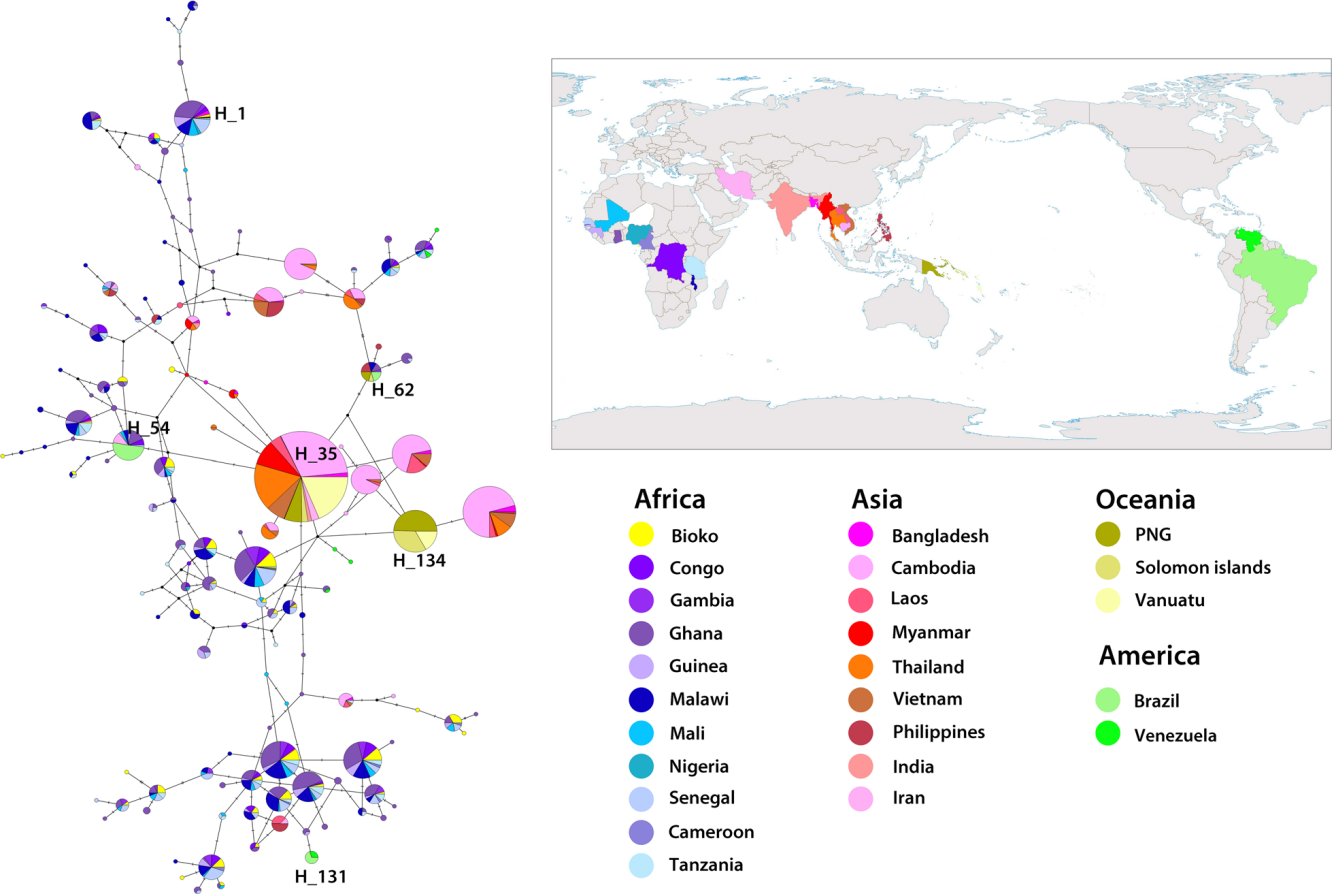
Haplotype network of C-terminal region among global *pfcsp*. Isolates from four continents and Bioko Island were marked in five different series colours, blue series for Africa, red series for Asia, khaki series for Oceania, green series for America, and yellow for Bioko Island

A haplotype network was constructed using 96 samples from Bioko in addition to 2200 global *pfcsp* C-terminal monoclonal sequences mining from the Pf3k database and NCBI (Fig. 5). The 2296 *pfcsp* C-terminal sequences were clustered into 138 unique haplotypes (H_1 to H_138). Detailed information of haplotypes was presented in Additional file 4. Fifty-eight haplotypes were shared by *pfcsp* sequences from at least two different countries; 70 haplotypes were limited to singleton (only composed by 1 sequence). And as for the H_1, which belongs to the 3D7 standard isolate, as well as the component of RTS,S malaria vaccine, only hold 2.08% (2/96) in Bioko isolates and 3.35% (77/2296) in the worldwide isolates, among which 74 isolates were found in Africa. Only H_62 was composed of samples from four continents (Africa, Asia, America and Oceania) but in a low prevalence (24/2296). Interestingly, the isolates from Africa and America shared the same haplotypes or the related ones (H_54, H_131), while the haplotypes of Oceanian isolates (H_35, H_134) have closer relationship with Asian’s. These phenomena correspond to the Fst index results shown in Table 3. As the Table 3 shown, Fst between Bioko Island and African mainland showed no significant population differentiation (Fst = 0.00878, p < 0.05). Meanwhile, clear population differentiation was identified between American, Asian, Oceanian and African parasite population (p < 0.05). Relatively closer genetic relationships were found in African & American parasite population and Asian & Oceanian parasite population (Fst = 0.19194, p < 0.05 and Fst = 0.06564, p < 0.05, respectively).

**Table 3:** Population pairwise fixation index (Fst) result

	Africa	America	Asia	Oceania
America	0.19194*			
Asia	0.24167*	0.21294*		
Oceania	0.25712*	0.42132*	0.06564*	
Bioko	0.00878*	0.24014*	0.31435*	0.42889*

* p < 0.05

## Discussion

Bioko Island, Equatorial Guinea, is a historically high malaria transmission region [16, 40]. Though BIMCP had launched in Bioko Island since 2004 and achieved a remarkable result, malaria is still a major health problem in this region. The genetic diversity and natural selection were analysed in Bioko *pfcsp* and global *pfcsp*. In general, the polymorphism patterns between Bioko *pfcsp* and African mainland *pfcsp* have no obvious differentiation, although the geographic location of Bioko Island was relatively isolated. This result might be explained by the work of Guerra et al., which reported that the strong connection of human movement between Bioko and the mainland Equatorial Guinea (EG), determine a high vulnerability of Bioko to malaria importation; these studies reported that the odds of malaria infection in travellers who had been to mainland EG were more than three times the rest of the population, which confirmed that the majority malaria cases are actively imported by off-island travellers to mainland EG [41, 42]. Furthermore, it is worth mention that the PfSPZ vaccine had been tested in Malabo and a series of clinical trials are undergoing, which might likely to affect the genetic background of the malaria parasites in this region [9]. According to the report [9], PfSPZ vaccine could induced the immune response to PfCSP, which might influence the genetic diversity and natural selection of *pfcsp* in Malabo. The natural selection analysis revealed that Bioko *pfcsp* might under a selection effect although there is no statistical significance (*p *> 0.1). These findings were in line with the prior studies about *P. falciparum* merozoite surface protein-1/2 (*PfMSP*-1/2) and *P. falciparum* apical membrane antigen-1 (*PfAMA*-1) genes in Bioko Island [43, 44].

N-terminal region of PfCSP plays an important role in the procedure of sporozoite invades to the hepatocytes [45]. In Bioko and global *pfcsp*, the genetic polymorphism of N-terminus was in a relatively low level. 19 amino acids length insertion and A98G were universally popular while several novel mutations were found with low frequency. Some scientists verified previously that the antibodies against to N-terminal region could be produced by host immune system and could evoke a partial inhibition of sporozoite invasion of hepatocytes in vitro [46]. Now the evidences of relatively conservative N-terminus might raise the possibility that whether the N-terminus has the potential to be a component of anti-malarial vaccine.

Central repeat region is an immunodominant epitope of PfCSP, and it had been applied to the component of RTS,S malaria vaccine [47]. Different numbers of tetrapeptide repeat was an important cause of *pfcsp* polymorphism. As expected, this study revealed the diversity of the number of tetrapeptide repeat (NANP/NVNP). Through the analysis among global different geographic regions, it was found that majority of samples possessed the tetrapeptide repeat ranging from 39 to 44 times. Though some scientists hold the view that the various number of tetrapeptide repeat make no significant impacts on RTS,S vaccine efficacy [14], it was known to correlated with the stability of CS protein structure [48]. However, the mechanism and effect of this variation is still unclear. For the universality of this variation, deeper research towards to this region is still necessary.

In the analysis of C-terminus of *pfcsp*, there were abundant polymorphisms found, especially in the TSR region (including Th2R and Th3R), the proven T cell immunogenic epitopes. The C-terminus of African, Asian, American and Oceanian samples presented their own distinctive diversity patterns. Not surprisingly, more polymorphisms were performed in the two larger-size parasite population (African and Asian) compared to those of America and Oceania. Because of the geographical isolation effect, some mutations showed the regional difference, for example the mutant at 325 position (N325Y) was only occurred in Asian countries; S326A was only found in Venezuela; wild type A361 was mainly observed in Africa, and so on. These phenomena indicated us that continuous monitor to these regional characteristic mutations, and exploration on their association with regional malaria epidemic situation are necessary.

In terms of C-terminal haplotypes analysis, 29 of 34 Bioko *pfcsp* haplotypes were shared with African continent samples while only 5 were limited to singleton, which implied that Bioko *pfcsp* was not completely independent of African continent. An obvious phenomenon was found that haplotypes from Oceanian *pfcsp* have closer genetic relationship with Asian haplotypes. Additionally, the same phenomenon happened among the parasites from America and Africa. It reflects that worldwide genotype of *pfcsp* C-terminus might divide into two major groups (Africa & America and Asia & Oceania), which probably caused by the frequent communication due to geographical advantages. It provides an insight of the vaccine design based on PfCSP that the regional differentiation might be took into consideration.

The absence of 3D7-matched *pfcsp* was not the uncommon finding anymore [13, 49]. Unsurprisingly, in Bioko Island, only 2% 3D7-matched *pfcsp* were found. A study about genetic diversity and protective efficacy of the RTS,S/AS01 malaria vaccine stated that the 3D7-mismatched malaria might probably weaken the efficacy of vaccine, especially the mutations at 299, 301, 317, 354, 356, 359 and 361 amino acid position [14]. In this research, the polymorphism situation of these loci showed different degrees. It is worth mentioning that mutation rate of position 317 reached 91% and mutation rate of position 361 reached 73%. As these mutations are so common and probably affect the vaccine effect, a question raised that whether these high-frequency alleles instead of the wild-type ones could be applied in the vaccine component.

In terms of the distribution of mutations, all the 66 mutations found from global sequences were located at CD8+ T cell epitopes, while 28 of them were located at the overlap of CD8 + and CD4+ T cell epitopes. It is well known that CD8+ and CD4+ T cell are thought to play a role in natural and sporozoite vaccine induced immunity in *P. falciparum* malaria [50, 51]. This raises the question of whether these mutations affect host immunity. Mutation-effect prediction of these 28 mutations showed that more than half of them were predicted as damaging (15 of 28). Notably, when mutations located at some specific positions (including the probably harmful position 317 and 354) [14], great changes have taken place on the free energy difference, which would result in destabilization on CS protein structure in difference extent. However, the specific mechanism of whether and how these mutations do harm to vaccine efficacy are still not clear. Therefore, continuous monitoring on these mutations and deeper exploration on the mechanism is still necessary.

According to this study, there are several new insights might be considered in the design and improvement of PfCSP-based vaccine: (1) The globally high frequency alleles instead of the wild-type ones of C-terminus might be used for composing vaccine. (2) The immunogenic and conservative N-terminus might be applied in the composition of vaccine. (3) The regional differences should be considered in the improvement of universal malaria vaccine, mainly divided as Asia-Oceania region and Africa-America region.

## Conclusions

In this study, the genetic diversity of Bioko and global *pfcsp* was analysed. The genetic polymorphism of *pfcsp* was found to be universal. Besides this, significant geographical differentiation of *pfcsp* were found around the world, which could mainly be divided into Asia & Oceania group and Africa & America group. Meanwhile, the 3D7 isolate was rare to found worldwide. Some mutations which are located at T-cell epitopes might impair the PfCSP-based vaccine efficacy by using prediction tools. Findings in this study filled in missing data of Bioko *pfcsp.* A holistic view of global *pfcsp* polymorphism was presented in the article and provides more insight for the improvement of malaria vaccine design.

## Supplementary information

**Additional file 1.** Ethical approval letter (Spanish version)

**Additional file 2.** Ethical approval letter (Chinese version).

**Additional file 3.** Global pfcsp sequences acquired from NCBI.

**Additional file 4.** Detail information of haplotypes.

## Data Availability

The datasets supporting the conclusions of this article are included with in the article.

## Abbreviations

BIMCP: Bioko Island Malaria Control Project
dS: Synonymous
dN: Nonsynonymous
EG: Equatorial Guinea
HRM: High-resolution melting
PfCSP: *Plasmodium falciparum* circumsporozoite protein
PfMSP-1/2: *Plasmodium falciparum* merozoite surface protein-1/2
PfAMA-1: *Plasmodium falciparum* apical membrane antigen-1
PNG: Papua New Guinea
RDT: Rapid diagnostic tests
R: Recombination parameter
Rm: Minimum number of recombination events
SNPs: Single nucleotide polymorphisms
