# Supplementary figures and images for: Genetic polymorphism of Plasmodium falciparum circumsporozoite protein on Bioko Island, Equatorial Guinea and global comparative analysis
Source: Malar J. 2020 Jul 13;19:245. doi: 10.1186/s12936-020-03315-4 (PMC7359586; doi:10.1186/s12936-020-03315-4)

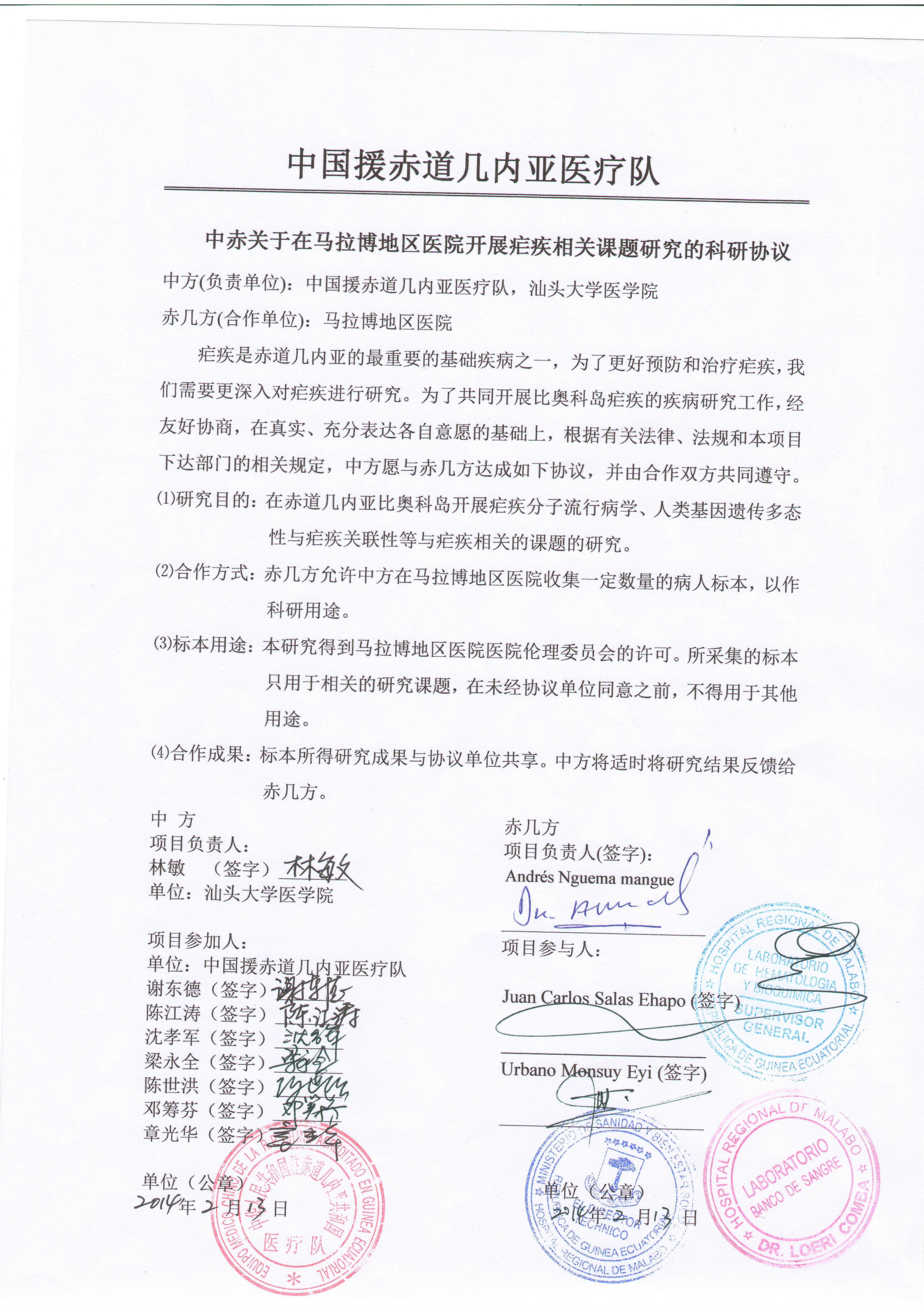

Supplement: Supplementary file 1 — Additional file 1. Ethical approval letter (Spanish version) [file 12936_2020_3315_MOESM1_ESM.jpg]

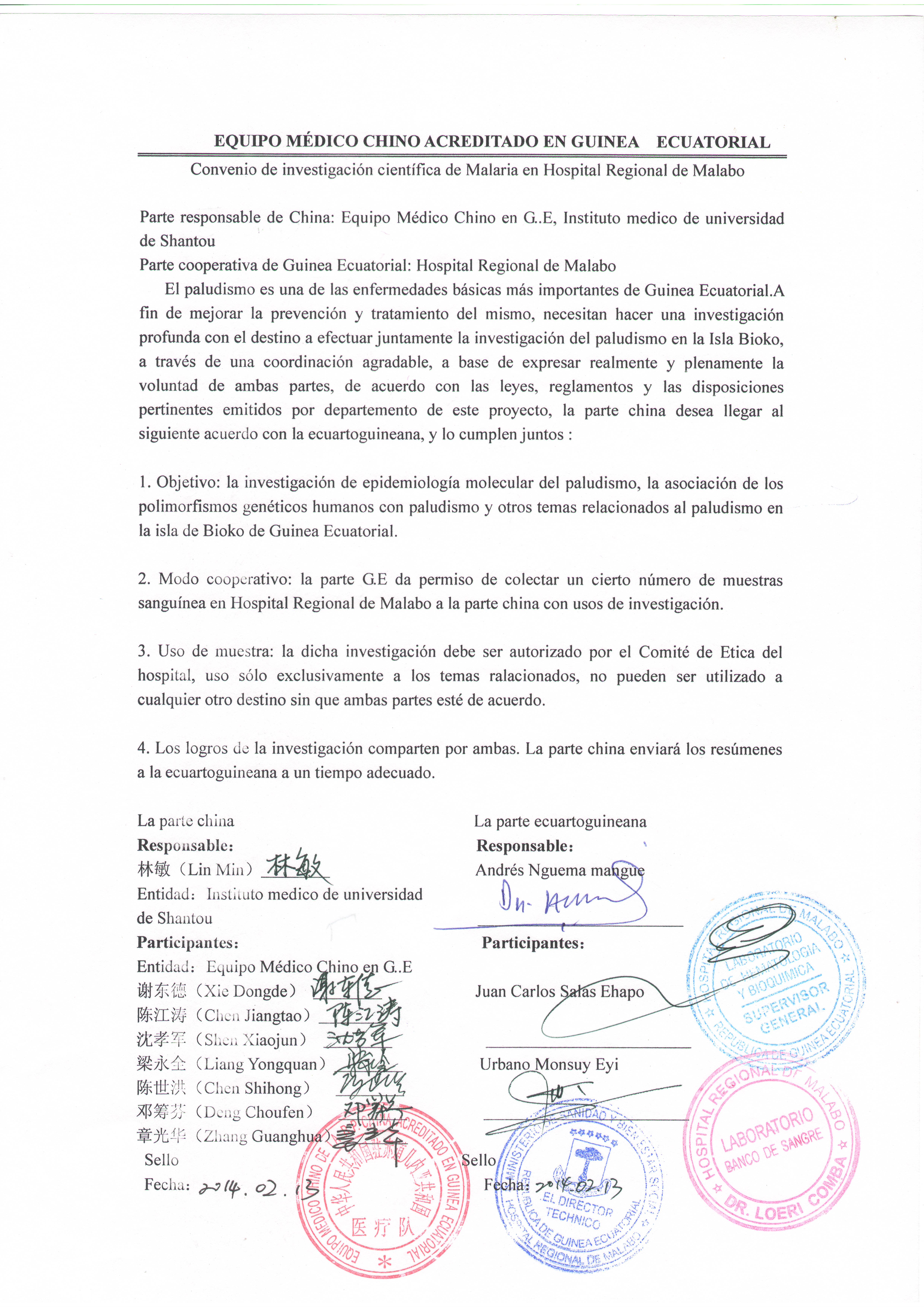

Supplement: Supplementary file 2 — Additional file 2. Ethical approval letter (Chinese version). [file 12936_2020_3315_MOESM2_ESM.jpg]
