# Supplementary material for: Genetic polymorphism of Plasmodium falciparum circumsporozoite protein on Bioko Island, Equatorial Guinea and global comparative analysis
Source: Malar J. 2020 Jul 13;19:245. doi: 10.1186/s12936-020-03315-4 (PMC7359586; doi:10.1186/s12936-020-03315-4)
Supplement: Supplementary file 3 — Additional file 3. Global pfcsp sequences acquired from NCBI. [file 12936_2020_3315_MOESM3_ESM.docx]

| **Country** | **Accession number** | ***n*** |
| --- | --- | --- |
| Philippines | AB502965− AB503006 | 42 |
| Iran | DQ521732− DQ521752 | 21 |
| India | AF540441, AF540442, AF540481-AF540486, AF540488 | 9 |
| Papua New Guinea | AB503007− AB503100 | 94 |
| Vanuatu | AB715520− AB715635 | 116 |
| Solomon Islands | AB503101− AB503151 | 51 |
| Cameroon | AF540444− AF540446, AF540449, AF540452, AF540453, AF540454, AF540468, AF540480 | 9 |
| Tanzania | AB502796− AB502855 | 60 |
| Venezuela | AF540458− AF540460, AF540466, AF540469, AF540470, AF540471, AF540478, AF540479 | 9 |
| Brazil | AB503152− AB503193 | 42 |

**Table S1** Global *PfCSP* sequences acquired from NCBI.
